# Genome-Wide Signatures of ‘Rearrangement Hotspots’ within Segmental Duplications in Humans

**DOI:** 10.1371/journal.pone.0028853

**Published:** 2011-12-14

**Authors:** Mohammed Uddin, Mitch Sturge, Lynette Peddle, Darren D. O'Rielly, Proton Rahman

**Affiliations:** Faculty of Medicine, Discipline of Medicine and Genetics, Memorial University, St. John's, Newfoundland, Canada; Seoul National University College of Medicine, Korea

## Abstract

The primary objective of this study was to create a genome-wide high resolution map (i.e., >100 bp) of ‘rearrangement hotspots’ which can facilitate the identification of regions capable of mediating *de novo* deletions or duplications in humans. A hierarchical method was employed to fragment segmental duplications (SDs) into multiple smaller SD units. Combining an end space free pairwise alignment algorithm with a ‘seed and extend’ approach, we have exhaustively searched 409 million alignments to detect complex structural rearrangements within the reference-guided assembly of the NA18507 human genome (18× coverage), including the previously identified novel 4.8 Mb sequence from *de novo* assembly within this genome. We have identified 1,963 rearrangement hotspots within SDs which encompass 166 genes and display an enrichment of duplicated gene nucleotide variants (DNVs). These regions are correlated with increased non-allelic homologous recombination (NAHR) event frequency which presumably represents the origin of copy number variations (CNVs) and pathogenic duplications/deletions. Analysis revealed that 20% of the detected hotspots are clustered within the proximal and distal SD breakpoints flanked by the pathogenic deletions/duplications that have been mapped for 24 NAHR-mediated genomic disorders. FISH Validation of selected complex regions revealed 94% concordance with *in silico* localization of the highly homologous derivatives. Other results from this study indicate that intra-chromosomal recombination is enhanced in genic compared with agenic duplicated regions, and that gene desert regions comprising SDs may represent reservoirs for creation of novel genes. The generation of genome-wide signatures of ‘rearrangement hotspots’, which likely serve as templates for NAHR, may provide a powerful approach towards understanding the underlying mutational mechanism(s) for development of constitutional and acquired diseases.

## Introduction

Segmental duplications (SDs) or low-copy repeats are blocks of DNA>1 kbp in size which share a high level of sequence homology (>90%) [1]–[4]. The catalogue of SDs comprises approximately 5% of the human genome encompassing 18% of genes [1]–[4]. They are considered antecedents to the formation of copy number variants (CNVs) which comprise approximately 12% of the human genome and are responsible for considerable human genetic variation [2], [5]. Emerging evidence suggests that SD regions are frequently associated with known genomic disorders with the vast majority representing novel sites whose genomic architecture is susceptible to disease-causing rearrangements [5]. However, the complexity of their structural architecture in the human genome and, more importantly, their role in disease pathogenesis remains largely elusive.

There is a growing body of evidence suggesting the involvement of multiple events in the origin of genomic rearrangements such as non-allelic homologous recombination (NAHR), non-homologous end joining (NHEJ), fork stalling and template switching (FoSTeS), and microhomology-mediated break-induced replication (MMBIR) [6]–[8]. Although the origins of the aforementioned mechanisms are strongly associated with highly homologous regions residing outside of common repeat elements (e.g., transposons) [9], the non-random distribution of highly homologous regions within SDs that are susceptible to such mechanisms remain to be fully elucidated. Moreover, evolutionary conservation of these mechanisms complicates the identification of SD breakpoints due to differing levels of sequence homology.

Genomic disorders arising from microdeletions/duplications fail to be adequately explained by a single underlying event. The true contribution of NAHR, NEHJ, MMBIR and FoSTeS events to the origin of genomic rearrangement remains elusive, although large-scale studies are beginning to implicate NAHR as one of the primary evenst contributing to the origin of these genomic copy number changes [9], [10]. Genomic DNA situated between distal and proximal SDs represents a critical region often reported to be deleted/duplicated due to misalignment of the SDs between homologous chromosomes [11]. Evidence suggests that the breakpoint architecture of SDs (i.e., distal and proximal) is associated with a higher propensity for NAHR-mediated rearrangement predisposing to an abnormal phenotype [12]. In other words, the increased frequency of pathogenic rearrangements is often directly correlated with the structural complexity of the local genomic regions involved. This is consistent with numerous reports indicating that highly homologous regions within SDs influence NAHR-mediated rearrangement events [9], [10], [13]. Throughout this paper, these highly homologous regions will be referred to as ‘rearrangement hotspots’. Classic examples of NAHR-mediated genomic rearrangement include genomic disorders such as 3q29 microdeletion/duplication syndrome, globozoospermia, and Williams-Beuren syndrome [14]–[16].

**Figure 1:**
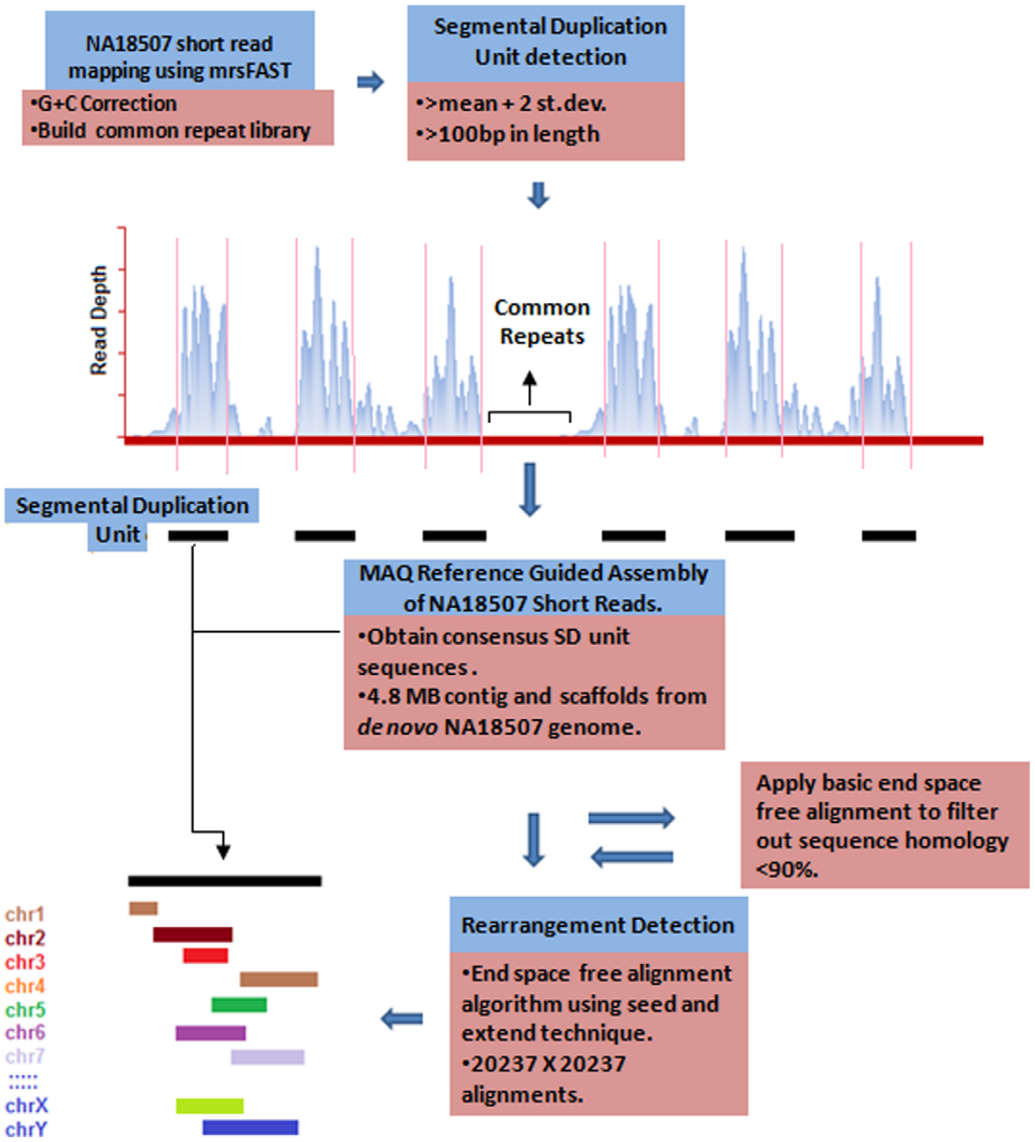
A schematic illustrating our hierarchical approach. mrsFAST was used to obtain read depth distribution of the NA18507 human genome with maximum mismatch (*n* = 2) was allowed against the repeat masked reference human genome (build 36). A mean-based approach was utilized to computationally predict the boundaries of regions associated with excessive read depth. MAQ was used to obtain the consensus genome (mapping quality *Q*>30 and *n* = 2) from the NA18507 genome assembly. The consensus sequence for highly excessive read depth regions was obtained in order to apply a window-based alignment algorithm. The previously identified novel 4.8 Mb sequence from de novo assembly within this genome was also included in the rearrangement analysis.

In a recent report, the detection and validation of 8,599 CNVs using microarrays [17] and subsequent targeted sequencing on 1067 of these CNV breakpoints [9] revealed extreme homologous regions consistent with NAHR-mediated rearrangements as the primary event in the origin of CNVs. In this study, we identified genome-wide ‘rearrangement hotspots’ within SD regions that often predispose to genomic disorders in humans, mediated predominately by NAHR. We specifically devised a hierarchical approach to detect SD units using an all-hit mapping algorithm, interrogating every 100 bp (GC-corrected read depth window with a 1 bp overlap) excluding common repeat elements. Reference-guided assembly was obtained from reads based on the NA18507 human genome and duplicated sequences were extracted from the assembly using detected breakpoints (**Fig. 1**). The primary objective of this study was to create genome-wide signatures of ‘rearrangement hotspots’ which can facilitate the detection of genomic regions capable of mediating *de novo* deletions or duplications in humans. To create a genome-wide high resolution map of ‘rearrangement hotspots’, we developed an end-space free pairwise alignment algorithm integrating a ‘seed and extend’ technique which can accurately investigate the complex structural architecture associated with the technically challenging and problematic nature of segmental duplications. The hypothesis of this study is that highly homologous SD regions (i.e., rearrangement hotspots) predispose to genomic rearrangements arising from recombination and replication-based events.

## Results and Discussion

### Detection of Segmental Duplication (SD) Units

Given that SDs intuitively consist of common repeat elements, SDs were fragmented into multiple smaller SD units which did not overlap with known repeat elements during the read depth-based analysis. In this study, 20,237 non-redundant sets of SD units with at least one inter- or intra-chromosomal rearrangement event were identified, representing 16.65 Mbp of SD units residing outside of common repeat elements in the human genome (**Information S1**). At first glance, this total content of SDs may appear small compared with that previously reported [3] and that reported in the database of genomic variants (DGV) which is mainly attributed to methodological differences (i.e., exclusion of common repeats, GC-correction, shorter window length, low read depth threshold). Results from this study and Perry *et al* [18], suggest that previously reported SD breakpoints are overinflated in size, further emphasizing the importance of creating a high-resolution map of ‘rearrangement hotspots’. Read depth distribution for duplicated and non-duplicated regions throughout the genome produced a distinctive distribution pattern with an approximate 7% error rate (**Information S1**).

Considering CNVs have a tendency to overlap with nearby SD breakpoints, the results of this study were compared with a recent study which identified common CNV breakpoints in three populations (i.e., 57 Yoruba, 48 European and 54 Asian individuals) [19]. The detected autosomal SD units greater than 200 bp shared 82% concordance (i.e., >50% overlap) with common CNV breakpoints using low coverage short-read data (**Information S1**). Moreover, 79% of breakpoints residing within genes with >3 copies as previously reported [20], were located within SD breakpoints identified in this study (**Information S1**).

Comparison with previous read depth-based reports highlights the advantages of our hierarchical strategy which include: 1) the use of a 100 bp read depth window with a 1 bp overlap to detect SD units which enabled the capacity to detect SD units with higher resolution; 2) the use of a lower threshold (i.e., mean +2 standard deviations) than previously reported methods in order to detect homozygous and hemizygous duplications; 3) fragmentation of SDs into smaller SD units in order to separate duplicated regions from common repeated elements while reducing alignment bias for rearrangement analysis and computational time; and 4) integration of end space alignment algorithm with a ‘seed and extend’ clustering technique to the duplicated region of the reference guided assembly sequences to perform an exhaustive search (i.e., 409 million alignments) to identify rearrangement breakpoints (**Information S2**).

Compared with copy number gains identified using microarray analysis [17], sequencing data used in this study revealed that autosomal SD unit breakpoints overlapped 54% with copy number gains [17], which increased to 67% when compared with 43× coverage (**Information S1**) [19]. Discrepancies are attributed to methodical biases, as detection of structural variants can be specific to different methodical approaches and discrepancies between methods can be as high as 80% [4]. The rearrangement analysis within the novel sequence revealed multiple hits within the duplicated sequences (i.e., >90% similarity) that were previously uncharacterized (**Information S2**.).

### Characterization of Rearrangement Hotspots Within Segmental Duplications

**Figure 2:**
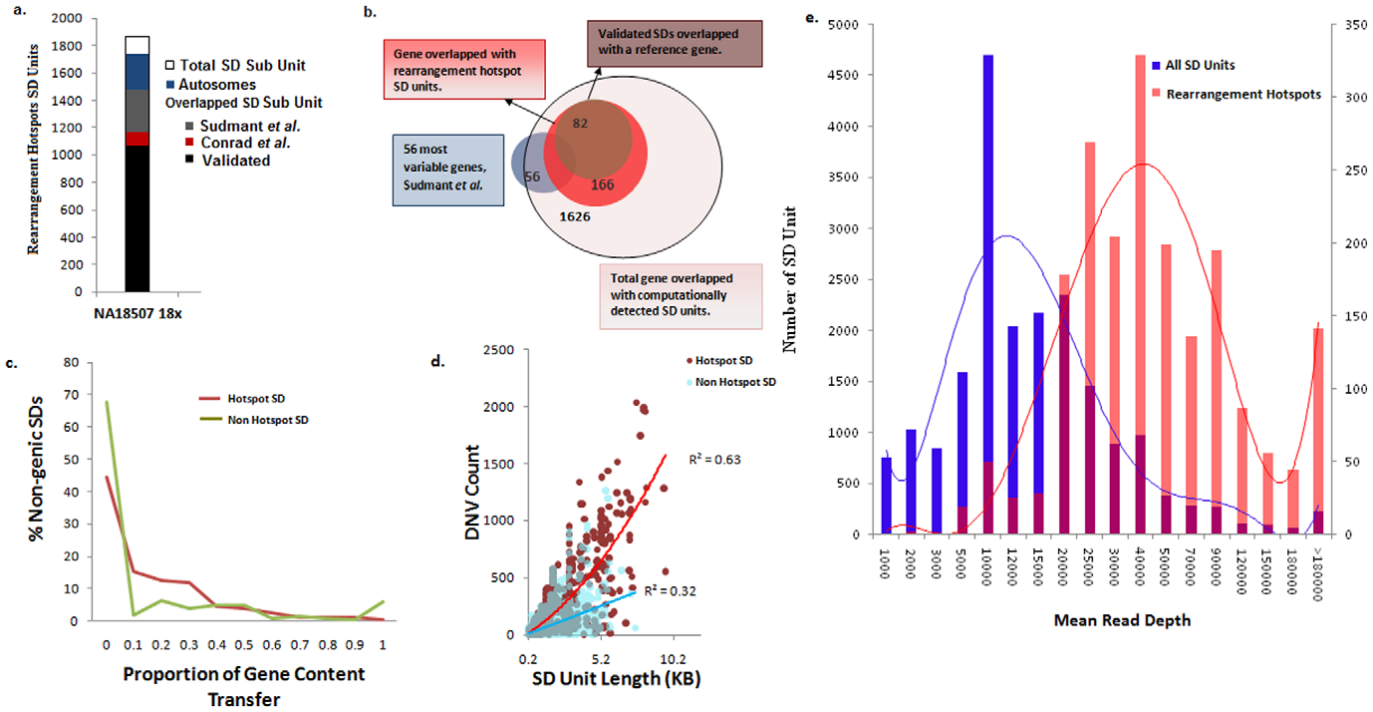
Segmental duplication (SD) units which represent the most complex rearrangements within the NA18507 human genome. **a**) A total of 1963 SD complex units (i.e., ≥10 rearrangements) were identified that were significantly different (p<1.0×10^−6^) compared with the rest of the NA18507 genome duplicated regions. The plot illustrates the concordance of the predicted autosomal complex regions compared with previous studies [17], [19]. **b**) Genes that completely or partially overlapped with detected SD units in which 73% (41/56) of the most variable genes in three different populations were detected in our analysis of the NA18507 human genome. Among the 1626 genes identified in this study, 10% (i.e., 166/1626) of genes that overlapped with a SD unit revealed extreme inter- and intra-chromosomal rearrangements, 50% of which have been previously validated [17]. **c**) Observed gene content transfer between hotspot and non-hotspot agenic SD units. **d**) scatter plot illustrating DNV count for hotspot and non-hotspot SD units. **e**) A histogram illustrating the mean read depth (RD) of the computationally predicted SD unit breakpoints. The blue bars represent the mean read depth for each of the 20,237 SD unit breakpoints and the red bars represent the mean read depth for hotspot regions.

Using 409 million pairwise alignments, we identified 1963 complex SD units or ‘rearrangement hotspots’ within SDs in the human genome with significantly high distribution of duplicons (*p*<1.0×10^−6^) with at least 10 duplicons per SD unit (**Fig. 2a**). Within these regions, an increase in copy number gain (i.e., increase of 62% in copy number gains within hotspots) with at least 50% overlap with SD units and CNV breakpoints has been observed compared with a previous report [17]. Importantly, 25% of these ‘rearrangement hotspots’ (i.e., 489/1963) overlapped with 166 unique genes (**Fig. 2b**) of which 77% (i.e., 375/489) were contained within 82 genes with increased copy number gain that have been previously validated using microarray analysis [17]. That 25 of these genes are highly variable in copy number within three populations indicates population-specific frequency of the underlying events in the origin of CNVs [19] which, in turn, implies an increase in frequency of genomic rearrangement events within hotspot regions. However, the extent of gene conversion within the NAHR hotspot is still unknown. In our analysis, we observed a relative increase of gene content transfer within agenic hotspot regions (i.e., approximately 50%) compared with the remainder of agenic non-hotspot duplicated regions (i.e., 32%) (**Fig. 2c**). The finding of elevated levels of gene content transfer is consistent with a previous study which hypothesized such a finding as an apparent feature for hotspots arising from homologous recombination [6]. Further analysis on duplicated gene variants (DNVs), which is a special type of paralogous sequence variant, was compared between the hotspot and non-hotspot duplicated regions [21]. We observed a 3-fold increase in DNVs located within hotspots compared with the remainder of the duplicated regions (*p*<0.0001) which implies greater diversity within hotspot regions. This finding is attributed, in part, to the accumulation of DNV-derived mutations among derivative homologous sequences within hotspot regions. We also observed a strong positive correlation (R^2^ = 0.63) between the length and the incidence of DNVs within hotspot regions (**Fig. 2d**). Genome-wide read depth comparison revealed that a subset of high read depth regions are positively correlated with rearrangement hotspots (**Fig. 2e**).

#### Distribution of Inter- and Intra-chromosomal Rearrangements

Segmental duplications (SDs) can be categorized according to the location of the rearrangement (**Information S1**) considering that recombination events can occur between homologues (i.e, inter-chromosomal) or by looping out within a single homologue (i.e., intra-chromosomal). Our analysis revealed that 7% of genes (i.e., 1,626/22,159) overlapped with 5,502 non-redundant SD units which represented 73% (i.e., 41/56) of the most highly variable genes previously identified in the human genome within three populations [19] (**Fig. 2b**). We have identified 91,971 duplicons (i.e., average of 4.5 duplicons per SD unit) with overlapping breakpoints throughout the SD regions. Extreme inter- and intra-chromosomal rearrangements occurred in 10% of genes (i.e., 166/1626) that overlapped with SD units, of which 50% have been previously validated [17]. Further analysis revealed that genic regions were enriched with intra-chromosomal recombination, whereas agenic regions evolved through both inter- and intra-chromosomal recombination (**Information S1**). Such intra-chromosomal recombination within genic SD units may represent conserved genomic organizations subject to gene conversion and concerted evolution [3], [6], [7]. Extreme variation, attributed in part, to SDs has been reported in at least 20% of the copy number variable gene families in three human populations [19].

Previous cytogenetic studies have demonstrated that pericentromeric and subtelomeric SD regions are strikingly polymorphic and both represent hotbeds for genomic rearrangement [22], [23]. Investigation of recombination within SD units revealed that pericentromeric regions of chromosomes 2, 5, 7, 10, 15, 16, 17, 22 and Y were enriched with inter-chromosomal recombination, whereas only chromosome 11 was associated with intra-chromosomal breakpoints (**Information S1**). Subtelomeric regions of chromosomes 1, 2, 4, 7, 9, 10, 11, 16, 19, 20, 22, and X were enriched with inter-chromosomal recombination, whereas chromosomes 3, 6, 12, 13, 14 and Y were associated with extreme intra-chromosomal breakpoints. This idiosyncratic rearrangement pattern suggests that multiple translocations involving distal regions of chromosomes create complex breakpoints within SDs. This is exemplified by the pseudoautosomal region 1 (PAR1) which displayed extensive inter- and intra-chromosomal tandem duplications, consistent with sex chromosome evolution (**Information S1**). Another complex region where extensive intra-chromosomal rearrangements were identified is the distal heterochromatic region of the Y chromosome (i.e., Yq*12*), housing the male specific (MSY) region (**Information S1**). A comprehensive map of this complex region was generated using PCR analysis in a previous study [24]. In our analysis, we detected both homozygous and hemizygous duplications using read depth information which represents an extension to previous SD analysis [19], [20] by the inclusion of sex chromosomes (**Information S1**).

An intriguing observation was the identification of complex rearrangements in multiple gene families where rapid evolution of *NBPF, PRAME, RGPD, GAGE, LRRC, TBC1, NPIP* and *TRIM* gene families appear to be predominantly attributed to intra-chromosomal gene transfer, whereas other complex gene families (e.g., *ANKRD, OR, GUSB, FAM, POTE, ZNF* and *GOLG*) appear to be more diverse with respect to transfer of gene content, occurring both within and between chromosomes (**Information S2**.). As previously reported [20], the *DUX* family gene was associated with the most copies within the reference genome. The rearrangement analysis of the novel sequence within 10q26.3 region suggests at least 10 additional copies of the *DUX4* gene is specific to novel sequences within the NA18507 human genome. (**Information S1**).

#### Gene Ontology Analysis within ‘Rearrangement Hotspots’

To investigate the impact of genes residing within ‘rearrangement hotspot’ regions identified in this study and their relation to complex disease, genes were functionally categorized using PANTHER gene ontology analysis (**Information S1**). Genes residing within ‘rearrangement hotspot’ regions appear to be involved in functions associated primarily with nucleic acid metabolism (22%) and cellular processes (16%), although associations also exist for developmental process (9%), cell cycle (9%), and cell communication (8%). This finding is consistent with a previous report in which copy number gains were associated with genes involved in nucleic acid metabolism and developmental processes, whereas copy number losses were enriched for genes involved in cell adhesion [25]. That genes residing in ‘rearrangement hotspot’ regions are consistently associated with functions affecting multiple processes important in normal growth and development, further underscores the critical role that rearrangement hotspots play in the genetic etiology of complex disease.

### Clinical Relevance of ‘Rearrangement Hotspots’

**Figure 3:**
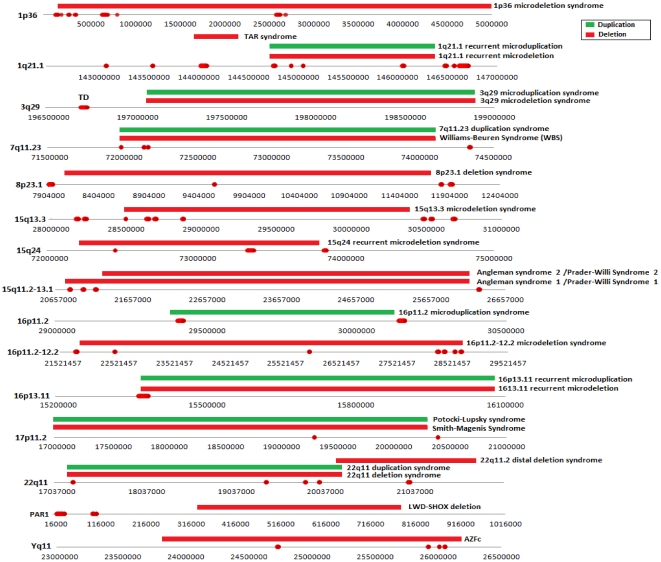
The physical position of rearrangement hotspots that has been mapped within the proximal/distal breakpoints of a pathogenic deletion (red horizontal block) or duplication (green horizontal block).

We have produced a genome-wide high resolution map of ‘rearrangement hotspots’ which likely serve as templates for NAHR and consequently may represent an underlying mechanism for development of constitutional and acquired diseases arising from *de novo* deletions or duplications. A collection of 24 previously identified genomic disorders predominantly mediated by *de novo* NAHR events are catalogued in the DECIPHER database [12]. Comparison of our hotspot regions with pathogenic deletions/duplications breakpoints mapped for those genomic disorders constituting only 15 common genomic loci revealed that 20% of the detected hotspots are clustered within proximal and distal SDs that are flanked by these pathogenic deletions/duplications (**Fig. 3**). This finding indicates a higher rate of NAHR within the genome-wide rearrangement hotspot regions detected in this study.

**Figure 4:**
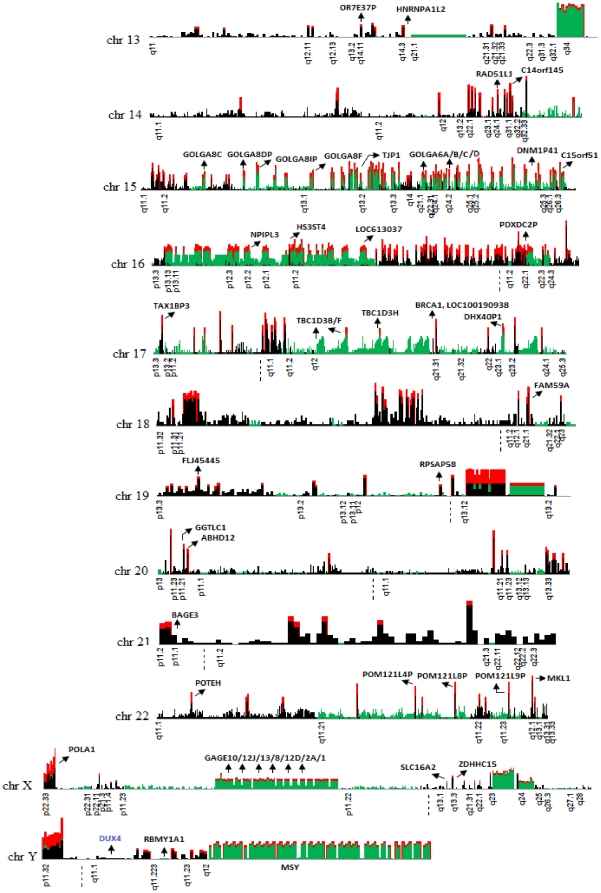
Landscape of chromosomal rearrangements in the NA18507 human genome. Chromosomal rearrangements located within duplicated regions are plotted against the human genome. Green bars represent the signature of intra-chromosomal rearrangements, black bars represent inter-chromosomal rearrangements and red bars represent ‘rearrangement hotspots’. Cytobands with duplications for each chromosome and selected genes that completely or partially overlapped with SD units are also indicated.

**Figure 5:**
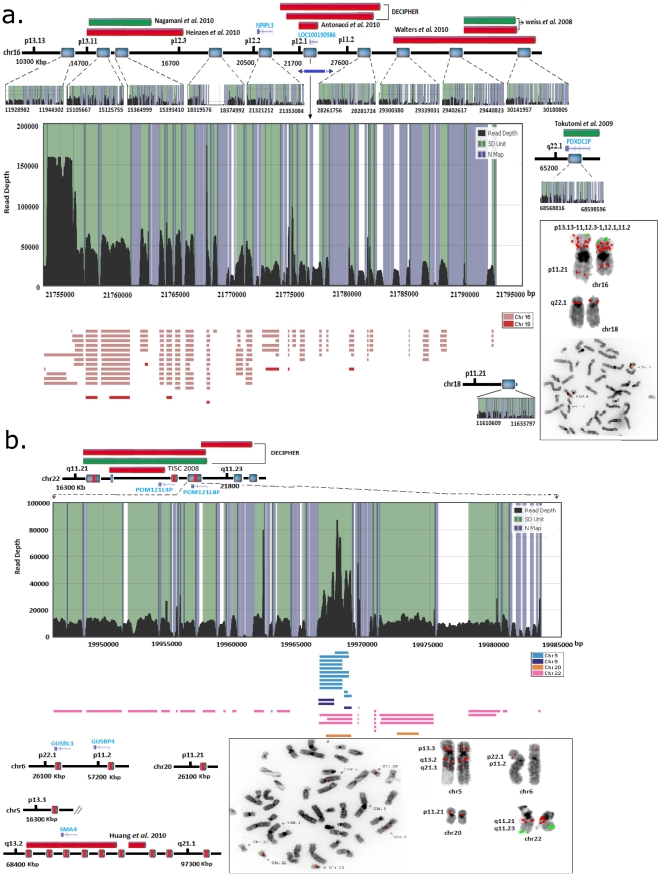
Signature of rearrangement hotspots located at a) 16p12.1 and b) 22q11.21. A 40 kbp region within 16p12.1 is illustrated with its corresponding derivative copies which were localized by hierarchical analysis. This region consists of the *NPIPL3* gene derivatives. The inter- and intra-chromosomal localization of the copies is approximated in the physical map within the chromosome contig (18p11.21). The alignments are color coded for chromosomes (i.e., color coded rectangles below the read depth plot) and FISH validation is illustrated for both inter- and intra-chromosomal localization. The pathogenic deletions and duplications located within these regions [27]–[32] are depicted in red and green bars, respectively The blue bars under the contig represent the approximated inversions previously reported by Antonacci, F. *et al* [26]. **b**) Analysis of a 37 kbp duplicated region within 22q11.21 revealed it is comprised of a core 2.7 kbp tandem duplicon copied from different chromosomes. Black lines represent the read depth (x-axis), green shade represent an SD unit, and blue bars represent the region with common repeat elements. The horizontal blocks (color coded according to chromosomes) are the rearrangement (intra/inter) fragments with >90% sequence similarity and >100 bp in length.

The rearrangement structure of these hotspots based on our *in silico* predictions (**Fig. 4**) reveals the complex architecture associated with SDs. To validate the complexity of these hotspots, FISH analysis was performed on selected regions harbouring hotspot clusters demonstrated 94% (i.e., 17/18) concordance with *in silico* predictions of co-localization (**Fig. 5a, 5b** **, and** **6**). One example of an identified ‘rearrangement hotspot’ is a duplication at the 16p12.1 complex region, which contains an S2 inversion [25], where the alignment localized multiple derivatives of the *NPIPL3* gene within chromosomes 16 and 18 (**Fig. 5a** and **Information S1**). The identified breakpoints revealed the presence of derivative copies of the *NPIPL3* gene within the short arm of chromosomes 16 and 18, possibly attributed to NAHR-mediated recombination, where pathogenic deletions and duplications have been reported in patients with mental retardation and intellectual disability [26]–[32]. The derivatives are located within the pathogenic deletion breakpoints among the patients with neurodevelopment disorders. Unfortunately, these studies used methodologies unable to localize derivative copies, and consequently the *NPIPL3* gene was disregarded as a susceptibility gene. A second complex region, 22q11.21, housed a large duplication consisting of two copies, with the ‘core duplicon’ being copied multiple times in chromosomes 5, 6, 20 and 22 (**Fig. 5b**). Phenotypes attributed to pathogenic deletions and duplications within chromosomes 5 and 22 [33], [34] revealed breakpoint patterns within a ‘core duplicon’, suggestive of NAHR-mediated duplication.

**Figure 6:**
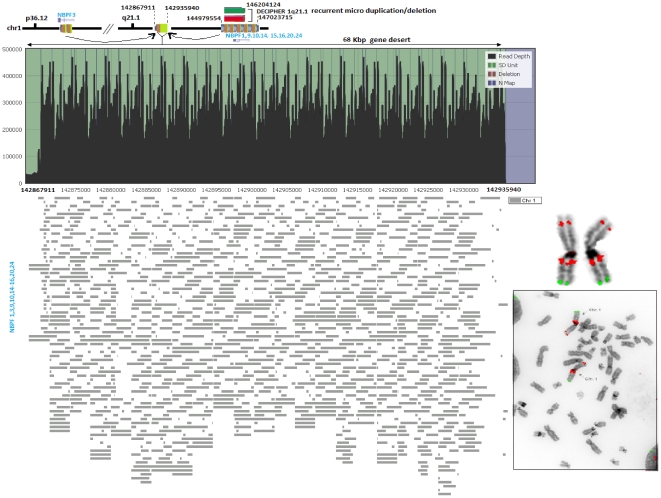
Rearrangement hotspots comprising a 68 kb gene desert located within 1q21.1 region. Validation of a gene desert where extreme intra-chromosomal rearrangement without any signature of inter-chromosomal duplication observed in our *in silico* predictions. The rearrangement consists of gene fragments from the *NBPF* gene family located within the p and q arm of chromosome 1.

A third complex region, revealed a previously uncharacterized gene desert within 1q21 indicating a possible harvest region for the *NBPF* gene family. This 68 Kbp gene desert region revealed extreme intra-chromosomal rearrangement without any signature of inter-chromosomal duplication in our *in silico* analysis (**Fig. 6**). The gene fragments from *NBPF1,3,9,10,14,15,16,20* and *24* appear to be copied and transferred to 1q21.1 (142867911–142935940) and consequently creating extreme overlapping tandem duplications. The fosmid clone G248P8712C10 covering this region was used on metaphase chromosomes to predict derivative duplicated loci. Multiple signals were obtained within 1p36.12 and 1q21.1 regions, while a weak signal was obtained within the 1p10p13 region which was not detected by our *in silico* analysis. The donor region located 2 Mb distal from the gene desert transferred gene content to this 68 kbp region which is associated with recurrent pathogenic deletions and duplications implicated in developmental disorders and neuroblastoma [12], [35], [36]. One may speculate that gene deserts may represent reservoirs for creation of novel genes and underscores the necessity to further explore this previously ignored region of the human genome. The complexity of tandem duplications (e.g., 1q21.1) can have a direct impact on estimating copy number for a gene (e.g., *NBPF*). In such cases, the estimation of copy number based solely on read depth may be affected due to the nature of the tandem duplication.

### Limitations

While the results of this study highlight the importance of restricting the number of vulnerable genomic regions that are targeted for clinical application, read depth-based approaches are associated with certain limitations. One of the limitations of our approach was the exclusion of inversions and insertions as the read map algorithm mrsFAST employed in this study was unable to return information regarding the orientation of duplicated loci [37] and as a result the map of ‘rearrangement hotspots’ will miss regions with complex orientations. Coverage is another constraint to detect SDs due to the positive correlation between coverage and detection rate [20]. A much higher (i.e., >40×) coverage will significantly increase the detection capacity of SD units. It is widely accepted that no single method has the capacity to capture the entire content of structural variants in the genome. For example, read pair and read depth approach overlapped only 20% among the detected variants [4], therefore, a portion of ‘rearrangement hotspots’ will be missed by our analysis. Moreover, a portion of highly duplicated regions (>99% sequence identity) analyzed in this study is reference sequence-specific due to MAQ's (mapping and assembly with quality) limitation to align short reads within those region precisely [38]. While the results of this hypothesis-driven *in silico* study are consistent with limited FISH analysis, additional genome-wide validation is required. In a recent report, it has highlighted the current limitation of *de novo* assembly approaches that produce a consensus genome with at least 16.2% shorter than the reference genome [39]. As *de novo* assembly progresses with large genome initiatives (i.e., 1000 genomes), integration of comprehensive *de novo* assembly with our hierarchical approach will afford maximum potential to detect a complete picture of population-specific ‘rearrangement hotspots’. Collectively, the results of this study emphasize the complexity of genomic rearrangements and the importance of NAHR-mediated recombination events in the origin of deletions and duplications which underlie the manifestation of germline and somatic disease.

Diseases arising from structural changes in the human genome are strongly correlated with the local sequence structure in which NAHR appears to be the predominant mechanism producing such vulnerable regions that often predispose to genomic diseases [6]. Isolating these regions based on high sequence homology will significantly reduce target regions and enable the development of hotspot-specific genotyping assays to capture disease associated deletions/duplication with both higher sensitivity and coverage. The breakpoints previously reported in SDs by aligning non-overlapping read depth windows of 5 kbp using the reference human genome [3], [20] limits the capacity to detect short highly homologous regions vulnerable to NAHR-mediated rearrangement. In this study, we have identified genome-wide ‘rearrangement hotspots’ with elevated frequency of pathogenic NAHR-mediated events. We have also detected an overwhelming number of overlapping CNV breakpoints, accumulation of DNVs and gene content transfer within hotspot regions. The read depth distribution of these hotspot regions revealed considerably higher read depth compared with the rest of the duplicated regions in the genome. The genome-wide characterization of ‘rearrangement hotspots’ will enhance the clinical applicability of high resolution genome analysis to uncover uncharacterized genomic disorders. Although current microarray platforms vary in both coverage and sensitivity [40], the generation of a genome-wide ‘rearrangement hotspot’ map will serve as a powerful tool for a custom design of microarrays targeting regions vulnerable to mutational events that predispose to genomic disorders.

Although NAHR appears to be the dominant mechanism in the origin of pathogenic chromosomal rearrangements, the complete identification of hotspot breakpoints due to NAHR, NHEJ, MMBIR and FoSTeS remains to be fully characterized. The generation of a genome-wide high resolution map of ‘rearrangement hotspots’, which likely serve as templates for NAHR, represents a risk factor for manifestation of constitutional and acquired diseases as these regions are capable of mediating *de novo* deletions or duplications. Fine mapping limited to only 20% of detected hotspot regions identified in this study using microarray will detect NAHR-mediated deletions/duplications for 24 known genomic disorders and the remaining 80% will increase the possibility of detecting novel *de novo* chromosomal loss or gain. We anticipate that discovery of genomic variants using this robust hierarchical approach will translate not into the replacement of microarray-based methods with whole-genome or exome sequencing of patients suspected to have complex disease. Instead, it represents a valuable tool which can be utilized for superior design and selection of probes, and ultimately the creation of a customized microarray chip specifically targeting ‘rearrangement hotspot’ signatures to detect complex genomic diseases.

## Materials and Methods

### Data Acquisition and Processing

We have obtained short read data for the NA18507 human genome sequenced using reversible terminator chemistry on an IIlumina Genome Analyzer [41]. The original data consisted of >30× coverage of the genome. We have obtained more than half of the data from the Short Read Archive Provisional FTP (NCBK) site (ftp://ftp.ncbi.nih.gov/pub/TraceDB/ShortRead/SRA000271/) with an average read length of approximately 36 bp. The analysis accuracy of this dataset has been previously described [41]. The 4.8 Mb novel sequence detected in the NA18507 genome by a previous *de novo* assembly was also integrated in our rearrangement analysis. The length distribution revealed that the contigs/scaffolds are over fragmented and >80% of the sequence length is <1 kb in length. The NA18507 human genome was selected as it is representative of the ancestral African Euroban population which has been previously shown to contain the most diverse polymorphisms compared with other populations [19], [20], rendering it an ideal sample to generate a ‘rearrangement hotspot’ map as the majority of the hotspot regions detected should exist within other populations.

### Short Read Mapping

We have applied mrsFAST (micro-read substitution only fast alignment search tool - version 2.3.0.2) which implements an all-to-all algorithm unlike other short read mapping algorithms [37]. Specifically, it is a fast alignment search tool which uses cache oblivious short read mapping algorithm to align short reads in an individual genome against a repeat masked reference human genome within a user-specified number of mismatches. We have mapped our short reads using mrsFAST with a maximum of two mismatches allowed against the repeat masked (UCSC hg18) genome assembly (**Information S1**). The advantage of using mrsFAST is that it returns all possible hits in the genome for a short read, allowing the detection of differential read depth distribution within duplicated regions of the human genome. Using the NA18507 human genome (18× coverage), 1.5 billion short reads were processed with 55.78% (i.e., ∼839 million short reads) mapped to the repeat masked human reference genome with the mrsFAST aligner (**Information S1**) which returned all possible mapping locations of a read; a key requirement to accurately predicting the duplicated regions within the reference genome.

### GC Correction

There exists a known bias with next generation sequencing technology towards GC-rich and GC-poor regions. Moreover, during library preparation using an Illumina Genome Analyzer, amplification artefacts are introduced in both GC-poor and GC-rich regions producing an uneven distribution of read coverage [20] which has the potential of detecting false positive duplicated regions. We have used a simple GC correction method to reduce this bias. Overlapping windows (i.e., by 1 bp) with length ‘*l*’ was used for read depth computation. Each read was assigned only once by its starting position and read depth was computed for each chromosomal position. The original mean read depth was calculated for each ‘*l*’ length (i.e., 100 bp) block using equation (1). We have computed G+C percentage for every 100 bp window from the reference human genome and the read depth was subsequently interrogated for adjustment. The adjusted read depth was computed using the following equation:
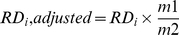
(1)where *RD_i_*, adjusted is the read depth after GC correction, *RD_i_* is the original read depth computed for *i^th^* window, *m1* is the overall median of all the windows with 100 bp length and *m2* is the mean depth for all windows with same GC percentage. All subsequent analysis was carried out on the GC-corrected read depth.

### Read Depth (RD) and Interval Detection

The first step in dissecting SD unit breakpoints using the NA18507 genome from all hit map information was to compute read depth from short read sequence mapping and detect SD intervals that do not overlap with a repeat region of the genome. Read depth was computed for each point after obtaining mapped anchoring positions of the short reads from mrsFAST. We have built a table for each chromosome, each containing coordinates where the common repeats are located. The read depth mean was computed for a chromosome from the genome content excluding common repeat regions. For each window with *l* length (100 bp) an event was determined. Events with excessive read depth and with a deletion were detected using equation (2).
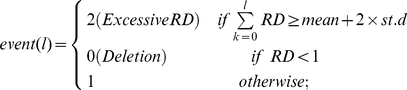
(2)


To investigate the interrogating window if it falls within a common repeat elements, we have built a library for the repeat masked regions (masked interspersed repeats, i.e. LINES, SINES, etc.) of the human genome. The mean length of the detected SD units was 822 bp (**Information S1**). The read depth distribution between the detected duplication subunits and the non-duplicated regions of the genome show significant read depth differences with an approximately 7% error rate (**Information S1**).

### NA18507 Short Read Reference Guided Assembly

The current version of mrsFAST does not return the quality of the aligned reads within a consensus genome. Instead, we have used MAQ version 0.7.1 (Mapping and Assembly with Quality) which assembles genomes with a specified quality. MAQ searches for the un-gapped match with lowest mismatch score (i.e., maximum of 2) in the first 28 bp. To confidently map alignments, MAQ assigns each alignment a Phred scaled quality score which measures the probability that the true alignment is not the alignment that is detected by MAQ. If a short read maps to multiple positions in the genome, MAQ will randomly pick one position and give the excluded position a mapping quality of zero. We have mapped and assembled the NA18507 genome short reads into the reference genome using MAQ allowing at most 2 mismatches.

### Detection of Genomic Re-arrangements

Using read depth as a measure to detect SD unit breakpoints may produce regions that share <90% sequence identity. To reduce false positive and computational burden after detecting SD unit breakpoints, we utilized a basic version of the end space alignment algorithm (without seed and extend approach) and performed pairwise alignment for each of the SD units against the rest of the genome SD units. We included only those SD units for rearrangement analysis described in the following section that contained at least one duplicon >100 bp with >90% sequence identity. We detected 20,237 SD units when every 100 bp window was assessed for a possible rearrangement.

### End-Space Free Alignment Algorithm

The ability to detect highly homologous regions between two sequences is essential for duplicon detection. Multiple clusters of non-adjacent duplicons with >90% sequence identity cannot be mapped using basic alignment algorithms. As previously reported, the basic pairwise global alignment algorithm will miss duplicon breakpoints that are non-adjacent within an SD with different thresholds of sequence identity [6]. Semi-global alignment has a tendency to produce pattern-like alignments (see example below), which are not informative for complex regions with multiple duplications. We have implemented a modified version of the pairwise alignment algorithm where the alignments are scored ignoring end spaces of the two sequences. Adding the option of end spaces in our alignment does not produce pattern-like alignments and therefore accurately pinpoints the breakpoints of the duplicon with an allowed gap that crosses the threshold of >90% sequence identity. The neutral rate of evolutionary decay suggests that 10% sequence divergence is required to accurately detect duplications that are primate-specific [6].

Example:

S1:ACGCAATTCGACTAGATCGGGTCGATGATCGATCGATGATCGAGACAGCATAGCAG

S2: CAATTCGACTAGATCGATCGACGATCGATCGAT

Semi-Global Alignment:

S1:ACGCAATTCGACTAGATCGGGTCGATGATCGATCGATGATCGAGACAGCATAGCAG

S2: ***CAATTCGACTAGATC*GATC***GA*CGATC***GAT*****C*G*AT*****

End-Space Free Alignment:

S1:CAATTCGACTAGATCGGGTCGATGATCGATCGAT

S2: CAATTCGACTAGATC*GATCGACGATCGATCGAT

In order to implement the algorithm, a dynamic programming technique was utilized which is a modified version of Smith-Waterman dynamic programming [42]. This approach will detect the pairwise alignment relative to a penalty function corresponding to semi-global alignment. We used the dynamic programming (DP) algorithm to compute the above alignments and used the backtrack pointer to identify the best alignment.

### Dynamic Programming Matrix and Recursive Trace Back

As a core searching algorithm, we have implemented a penalty function to complete the dynamic programming matrix *M*. First, we initialized the first column and row with zeroes which provided forgiving spaces at the beginning of the sequences in order to obtain the highest similarity between the interrogated sequences. Our intention was to locate duplicons between a pair of sequences (i.e.., s and t) with >90% identity and alignment with minimal gaps to avoid pattern-like structures. We encoded A with 1, G with 2, C with 3 and T with 4 to construct the 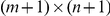 DP matrix *M*, where *m* and *n* is the length of two given sequences *s* and *t*, respectively. The algorithm uses a dynamic programming technique to fill a matrix *M* by a look up penalty function from the 5×5 matrix *C*. We have introduced penalty function *g(i,j)* for matched alignment with a score of 2. For the mismatches between a pair of bases, we introduced a penalty of −2 for mismatch and −3 for misaligned sequence produced by sequence assembly tools (i.e., MAQ). We used a −3 penalty to reduce the amount of misaligned portions of the sequence into duplicon identification. To allow the algorithm to ignore the end positions of the sequences if it has low similarity, we have performed a trace back from the highest value returned by function *Sim(s,t)* in the matrix *M* (**Information S1**). For any two given sequences (i.e., *s* and *t*), a semi-global alignment is an alignment between a substring (in this case duplicon) of *s* and *t*.
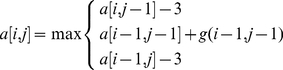
(3)

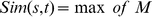
(4)


The memory requirement to fill out DP matrix *M* is *O(mn)*. The computational time to complete the dynamic programming Matrix *M* and to determine the maximum value in *M* for a given pair of sequence *s* and *t* with nearly similar length is *O(n^2^)* and to trace back starting from the maximum point in the matrix takes *O(m+n)* time to obtain optimal alignment.

Now, it might be apparent that ignoring end spaces might not detect true breakpoints and for long sequences it might produce really short alignments. Considering that majority of the commonly used alignment search methods (i.e., BLAST, BLAT, and SHRiMP) implement a “seed and extend” method to obtain faster sequence comparison [43]–[46], this method was also applied in this study. To perform an exhaustive search within the scope of 100 bp windows for any two given segmental unit sequences obtained from NA18507 genome, we have applied the dynamic programming algorithm for each 100 bp window with 10 bp overlaps as “seeds”. The highly similar seeds (>90%) went through the “extend” step and the rest was ignored. We acknowledge that this approach might detect the same breakpoints multiple times if multiple seeding events are obtained from a highly duplicated region. Therefore, we have compared the previously extended duplicon breakpoints from the same SD unit and the overlapping “seeds” and only the maximum extended duplicon was kept (**Information S1**). ‘Extend’ is a recursive procedure which extends bi-directionally by 10 bp and the extend step ceases in each direction when further extension does not cross the sequence identity threshold. As a result, the procedure terminates if any further extension of both directions returns <90% sequence identity.

### FISH Validation

Cytogenetic preparations were made from lymphoblastoid culture (obtained from Coriell cell repositories) for the NA18507 sample. The cell suspension was dropped on slides using a thermotone, aged overnight and hybridized with test (i.e., spectrum orange) and control probes. Following post-hybridization washes and 4,6-diamidino-2-phenylindole (DAP1) counterstaining, slides were analyzed using fluorescence microscopy. Pseudocoloring and image editing was performed using Photoshop software. To validate our duplicon rearrangement within SD units, we selected three complex regions in the human genome: 1q21.1, 16p12.1 and 22q11.21. In this study, we used fosmid genomic clones corresponding to a duplicated locus as a probe against chromosomal metaphase. The localization of FISH clones within these regions and the corresponding derivative loci validated >94% (i.e., 17/18) of the *in silico* co-localization predictions. The FISH technique was unable to provide a precise estimate of rearrangement at the level of 100 bp due to resolution limitations (**Information S1**).

### Permutation

The basic analyses were conducted using a permutation procedure to assess statistical significance of 1-sided tests. The rearrangement for each SD unit was permuted randomly between the two groups and test statistics was computed in each permutation. All results reported in this study used 1 million permutations to derive an empirical P-value.

#### Gene Ontology Analysis

Gene ontology data analysis was performed using PANTHER (version 7.0) database [45]. We have analyzed the biological processes of the hotspots genes (**Information S1**).

## Supporting Information

Information S1 **Consist of supplementary figures and tables.**
(DOC)

Information S2 **Segmental duplication unit breakpoints and their associated duplicons.**
(XLS)
