# Supplementary material for: Genome-Wide Signatures of ‘Rearrangement Hotspots’ within Segmental Duplications in Humans
Source: PLoS One. 2011 Dec 14;6(12):e28853. doi: 10.1371/journal.pone.0028853 (PMC3237539; doi:10.1371/journal.pone.0028853)
Supplement: Information S1 — Consist of supplementary figures and tables. (DOC) [file pone.0028853.s001.doc]

**Supplementary Figures and Tables for “Genome-wide signatures of ‘rearrangement hotspots’ within segmental duplications in humans”**

Mohammed Uddin, Mitch Sturge, Lynette Peddle, Darren D O’Rielly, Proton Rahman

Faculty of Medicine, Discipline of Medicine and Genetics, Memorial University, St. John’s, Newfoundland, Canada.

**^*^Address for all correspondence:**

Dr. Proton Rahman

Professor of Medicine

St Clare’s Mercy Hospital

1 South - 154 LeMarchant Rd

St. John’s, Newfoundland

Canada A1C – 5B8

Phone: 709 777 5732

Fax: 709 777 5212

Email:prahman@mun.ca

**List of Figures:**

Figure 1. Length and GC distribution of segmental duplication (SD) units detected by computational prediction………………………………………………………………… 3

Figure 2**.** Read depth distribution of the complete human genome segmented between duplicated and non-duplicated regions………………………………………………… 4

Figure 3. Population comparison of detected SD units…………………………….. 5

Figure 4. Concordance of autosomal SD unit in three populations are depicted in color-coded histograms……………………………………………………………………….. 6

Figure 5. Breakpoint comparison (i.e., >50% overlap) of highly variable genes…. 7

Figure 6. Criteria for inter- and intra-chromosomal rearrangements……………… 8

Figure 7. Inter- and intra-chromosomal rearrangement distribution for the NA18507 genome depicting the landscape of human genic and agenic region rearrangements with a 99% confidence interval………………………………………………………… 9

Figure 8. Rearrangement within PAR (pseudohomologous region)………………. 11

Figure 9. The localization of the *DUX* gene family………………………………….. 13

Figure 10. Localization of the *NPIP* and *NPIPL3* gene derivatives……………….. 14

Figure 11. End space free alignment algorithm. ............................................... 17

Figure 12. The ‘seed and extend’ mechanism to detect optimal seeds with a 100 bp window of alignment. ………………………………………………………………….. 18

Figure 13. Distribution of gene ontology categories…………………………………. 19

**List of Tables:**

Table 1. Rearrangement analysis of SD units within genic, subtelomeric and pericentromeric regions of the human genome……………………………………… 10

Table 2. Summary of FISH analysis………………………………………………….. 14

Table 3. Short reads map statistics…………………………………………………… 19

**References**…………………………………………………………………………. 20


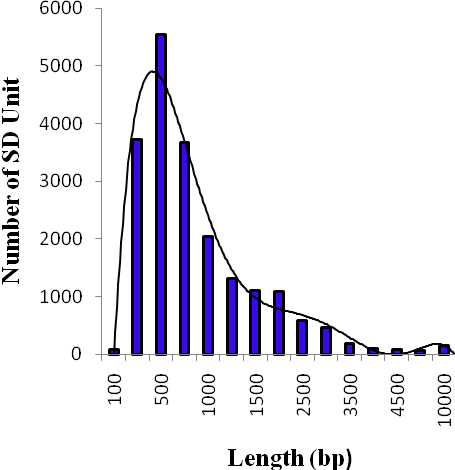

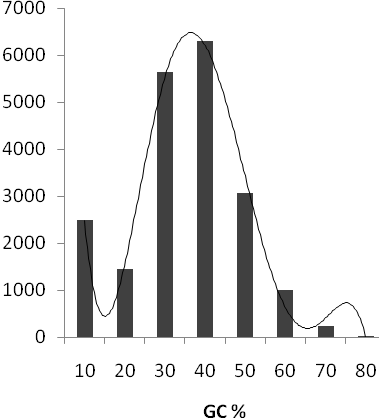


**Supplementary Figure 1.** Length and GC distribution of segmental duplication (SD) units detected by computational prediction. The mean size was 822 bp after exclusion of common repeat elements from duplicated loci (left Figure). Interestingly, the majority (i.e., approximately >80%) of these loci were located within the 30^th^ to 70^th^ G+C percentile (right Figure).

#
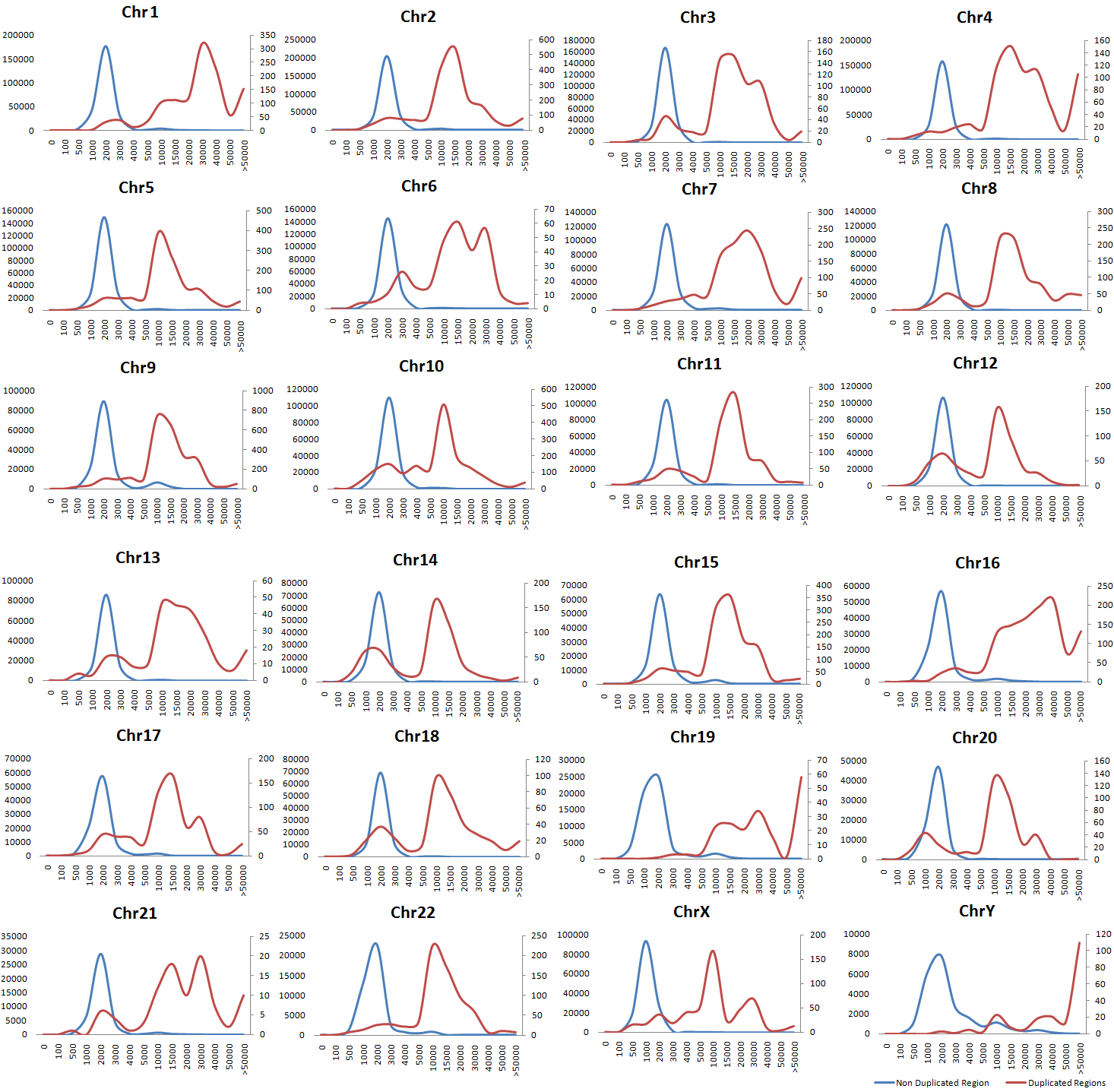


**Supplementary Figure 2.** Read depth distribution of the complete human genome segmented between duplicated (right Y-axis - red line) and non-duplicated regions (left Y-axis - blue line). The distribution illustrates a distinctive distribution pattern between duplicated and non-duplicated regions with an approximate 7% error rate. These discrepancies are largely attributed to micro-deletion polymorphisms that are located within the SD units as previously reported [1].

#
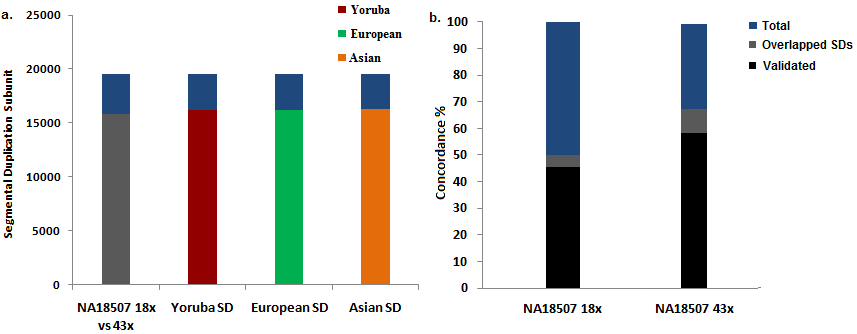


**Supplementary Figure 3.** Population comparison of detected SD units. **A)** The bar chart illustrates the comparison between detected duplicated regions which are common between 18x and 43x coverage of the NA18507 human genome. The SD regions detected using low coverage short read data in three different populations (i.e., 57 Yoruba, 48 European and 54 Asian individuals) illustrate a concordance of 82.95%, 83.03% and 83.19%, respectively. **B)** The first bar (NA18507 18X) represents a comparison between this study and Conrad *et al.* [2] study while the second bar (NA18507 43X) represents a comparison between studies by Sudmant *et al.*[3] and Conrad *et al*. [2].


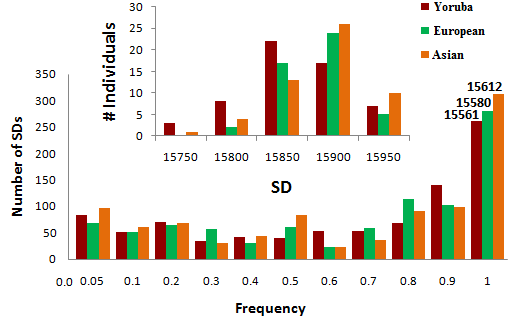


**Supplementary Figure 4.** Concordance of autosomal SD unit in three populations are depicted in color-coded histograms. More than 90% of the concordant SD units are common within these three populations. Note that the average read depth for most of the individuals varies from 1.5 to 7x. The Asian population is associated with a higher error rate which is attributed to the lowest coverage [3].


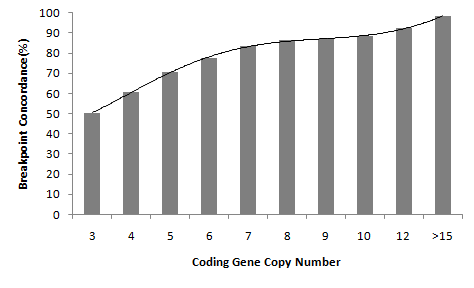


**Supplementary Figure 5.** Breakpoint comparison (i.e., >50% overlap) of highly variable genes detected by Alkan *et al.*[1] illustrates that 79% of these genes are within our detected segmental duplication (SD) breakpoints.


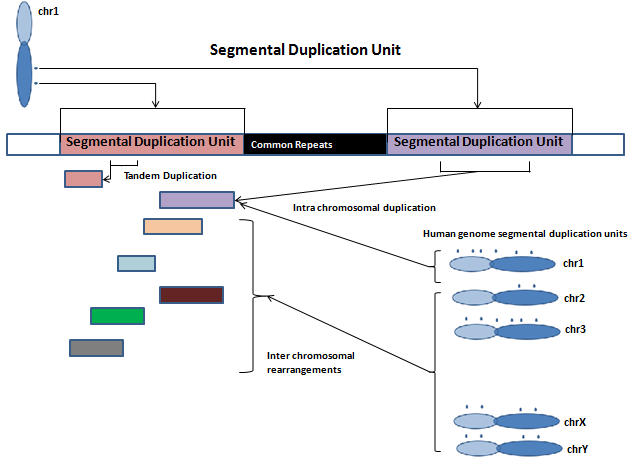


**Supplementary Figure 6.** Criteria for inter- and intra-chromosomal rearrangements. In this scenario, two mechanistic evolutionary forces are imposed (intra-chromosomal rearrangements including tandem duplications, and inter-chromosomal rearrangements) together to illustrate the criteria for rearrangements.


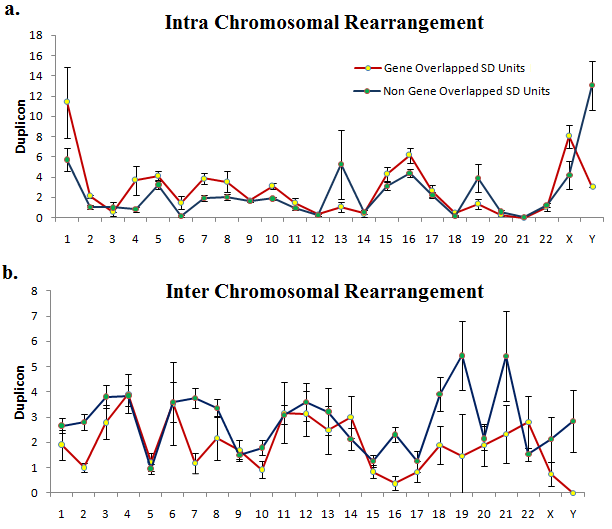


**
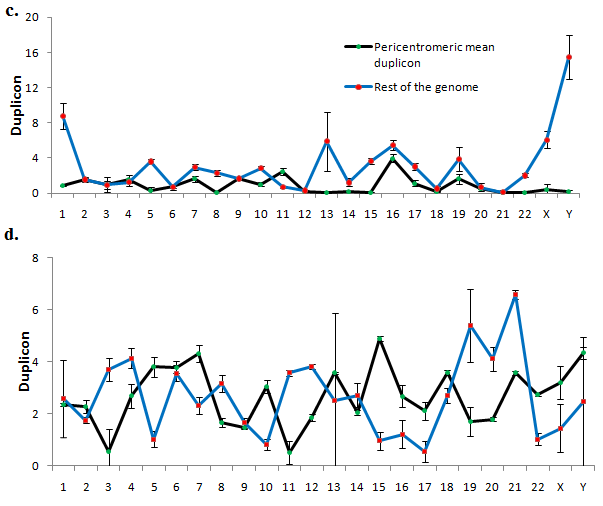
**

**Supplementary**
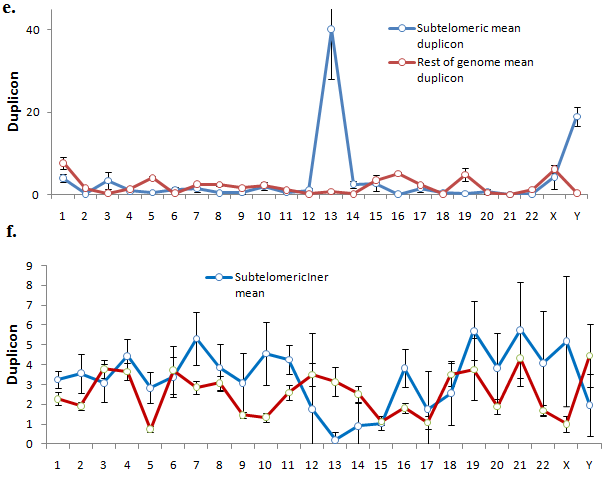
 **Figure 7.** Inter- and intra-chromosomal rearrangement distribution for the NA18507 genome depicting the landscape of human genic and agenic region rearrangements with a 99% confidence interval. **A)** Intra-chromosomal rearrangement enrichment and **B**) low inter-chromosomal rearrangement was observed within genic regions as compared with agenic regions. For Y chromosome the analysis shows no inter chromosomal rearrangement within the genic duplicated region (excluding DUX family region from the analysis). Mean duplicons that define pericentromeric duplications with **C)** intra-chromosomal and **D)** inter-chromosomal rearrangements. **E)** The mean duplicon within the telomeric region with intra-chromosomal rearrangement depicts chromosome 13q34 and Yq12 as an outlier with extensive intra-chromosomal rearrangement. **F)** Illustrates that inter-chromosomal rearrangement is dominant within the telomeric region of NA18507 human genome.

**Supplementary Table 1.** Rearrangement analysis of SD units within genic, subtelomeric and pericentromeric regions of the human genome.

| **Chromosome Genic / Non-Genic Subtelomeric Pericentromeric**  **Intra *P^Ŧ^* Inter *P^Ŧ^* Intra *P^Ŧ^* Inter *P^Ŧ^* Intra *P^Ŧ^* Inter *P^Ŧ^*** |
| --- |
| | **1** | 1.0 X 10^-6^ | 0.995 | 0.999 | 1.0 X 10^-6^ | 1 | 0.841 | | --- | --- | --- | --- | --- | --- | --- | | **2** | 1.0 X 10^-6^ | 1 | 1 | 1.0 X 10^-6^ | 0.479 | 3.5 X 10^-4^ | | **3** | 0.843 | 0.996 | 1.0 X 10^-6^ | 0.939 | 0.193 | 0.999 | | **4** | 1.0 X 10^-6^ | 0.407 | 0.974 | 9.5 X 10^-3^ | 4.8 X 10^-2^ | 0.999 | | **5** | 1.0 X 10^-6^ | 4.5 X 10^-2^ | 1 | 1.0 X 10^-6^ | 1 | 1.0 X 10^-5^ | | **6** | 1.0 X 10^-6^ | 0.526 | 3.3 X 10^-4^ | 0.695 | 0.234 | 0.405 | | **7** | 1.0 X 10^-6^ | 1 | 0.992 | 1.0 X 10^-6^ | 1 | 1.0 X 10^-6^ | | **8** | 8.0 X 10^-6^ | 0.996 | 0.999 | 3.3 X 10^-2^ | 1 | 0.986 | | **9** | 0.261 | 0.106 | 1 | 5.9 X 10^-5^ | 0.649 | 0.869 | | **10** | 1.0 X 10^-6^ | 0.999 | 0.766 | 1.0 X 10^-6^ | 1 | 1.0 X 10^-6^ | | **11** | 3.2 X 10^-3^ | 0.418 | 1 | 1.0 X 10^-6^ | 1.0 X 10^-6^ | 1 | | **12** | 0.166 | 0.827 | 1.0 X 10^-6^ | 0.770 | 0.985 | 0.999 | | **13** | 0.997 | 0.908 | 1.0 X 10^-6^ | 1 | 1 | 1.9 X 10^-2^ | | **14** | 0.784 | 9.9 X 10^-4^ | 1.0 X 10^-6^ | 0.990 | 1 | 0.955 | | **15** | 1.3 X 10^-5^ | 0.997 | 0.779 | 0.652 | 1 | 1.0 X 10^-6^ | | **16** | 1.0 X 10^-6^ | 1 | 1 | 1.0 X 10^-6^ | 1 | 1.0 X 10^-6^ | | **17** | 6.3 X 10^-2^ | 0.951 | 0.965 | 0.123 | 1 | 1.0 X 10^-6^ | | **18** | 3.2 X 10^-4^ | 0.999 | 2.9 X 10^-2^ | 0.865 | 0.999 | 8.4 X 10^-2^ | | **19** | 0.999 | 0.999 | 1 | 8.0 X 10^-3^ | 0.998 | 0.999 | | **20** | 0.999 | 0.714 | 9.2 X 10^-2^ | 5.6 X 10^-5^ | 0.832 | 0.999 | | **21** | 1 | 0.999 | 1 | 6.5 X 10^-2^ | 0.933 | 0.995 | | **22** | 0.865 | 9.0 X 10^-6^ | 0.998 | 1.4 X 10^-3^ | 1 | 1.0 X 10^-6^ | | **X** | 1.0 X 10^-6^ | 0.999 | 0.913 | 1.0 X 10^-6^ | 0.997 | 1.6 X 10^-2^ | | **Y** | N/A^*^ | N/A^*^ | 1.0 X 10^-6^ | 0.999 | 1 | 8.6 X 10^-3^ | |

**Note:** The SD units that overlap with a reference gene were analyzed for each chromosome based on the captured inter- and intra-chromosomal rearrangements against the rest of the human genome. The inter and inter-chromosomal rearrangement of the segmental duplications within subtelomeric and pericentromeric regions were also analyzed against the rest of the duplicated regions in the human genome.

Ŧ - The table shows a series of 1-tail analysis of genomic rearrangements using 1 million permutations of mean differences to obtain empirical *P*-values.

*Chromosome Y contained only two genes (i.e., *RBMY1A1* and *RBMY1F*) which revealed intra-chromosomal rearrangements, whereas the *DUX4* gene was associated with inter-chromosomal rearrangement. Thus, we have excluded chromosome Y from permutation test.


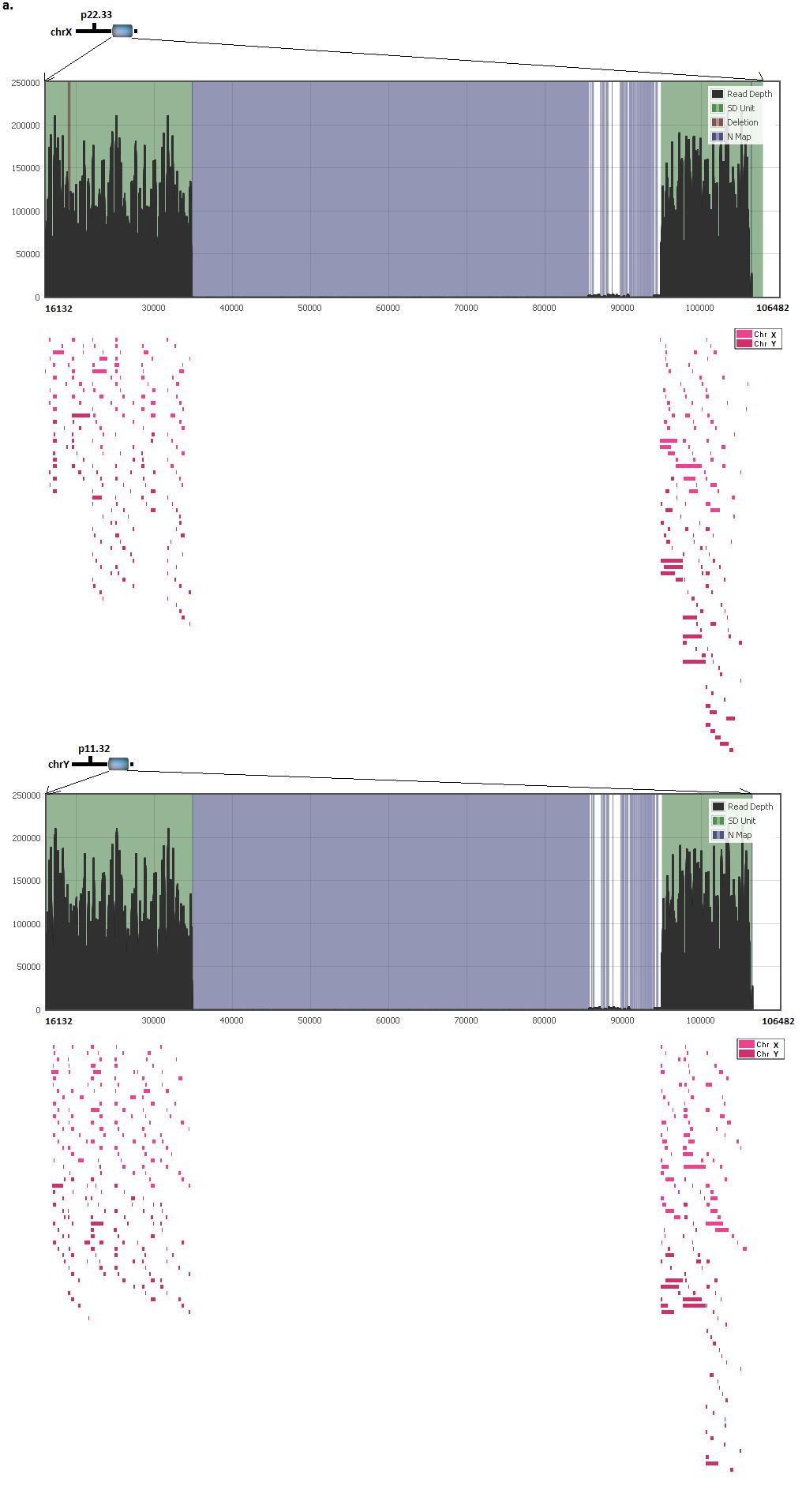


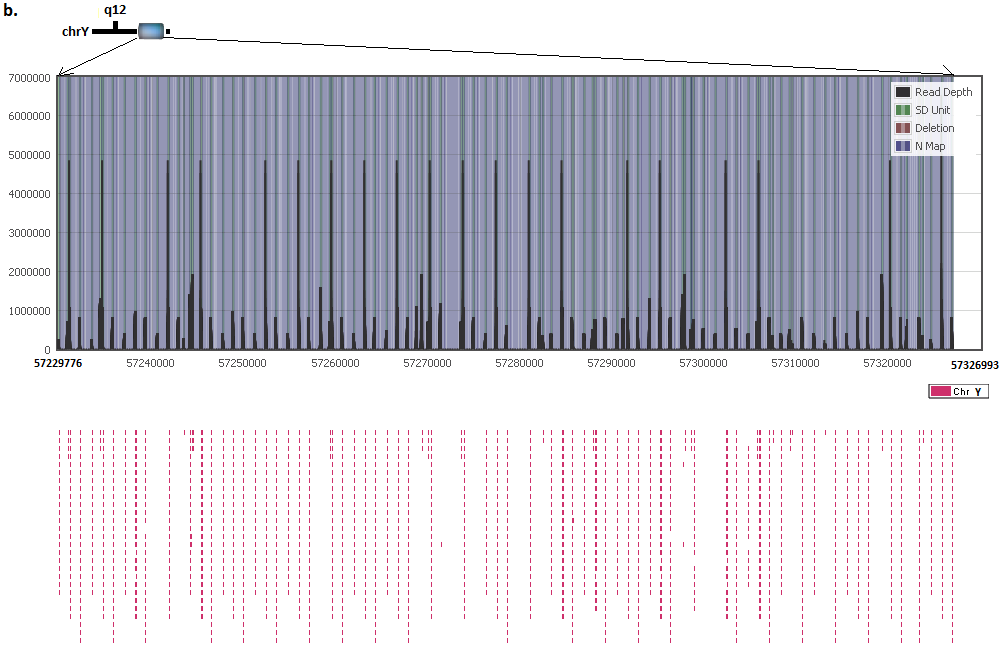


**Supplementary Figure 8.** Rearrangement within PAR1 (pseudohomologous region 1). **A)** The pseudohomologous region of the sex chromosomes contain a 90 kbp duplication between chromosomes X and Y where extensive inter- and intra-chromosomal tandem duplications were observed. **B)** A 90 kbp region within Yq12 locus illustrating extreme tandem duplication with a duplicon of 100 bp in length.

#
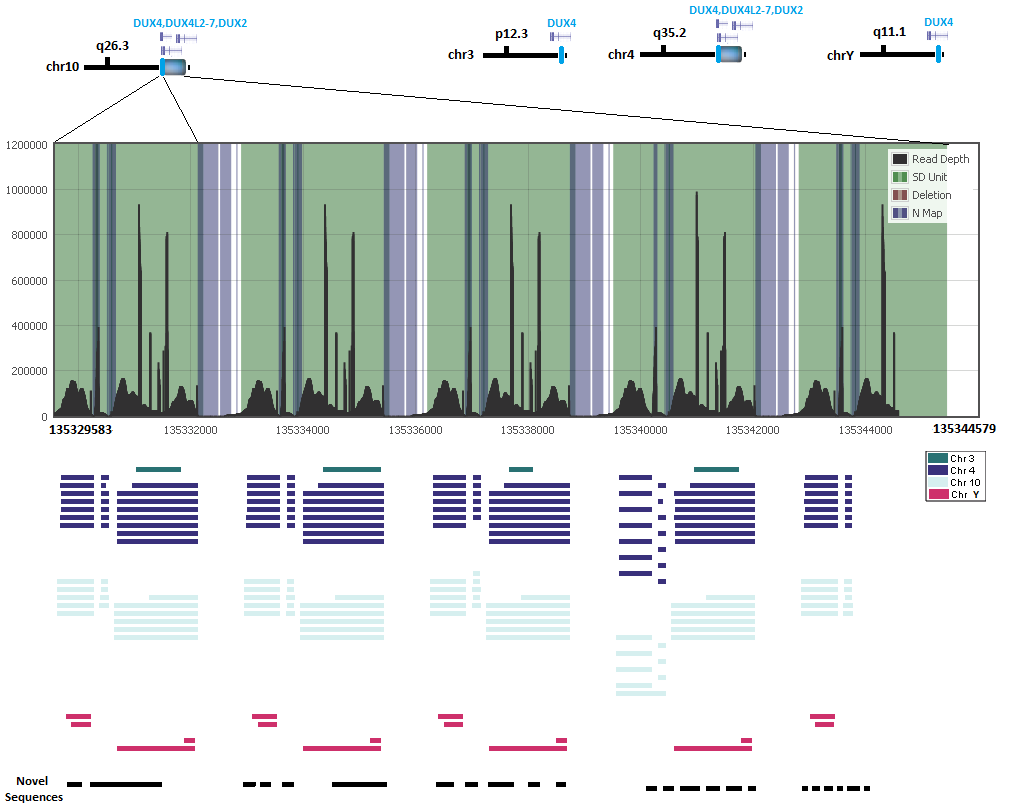


**Supplementary Figure 9.** Localization of the *DUX* gene family illustrating copies at the base pair level in chromosomes 3, 4, 10, Y and within the novel *de novo* assembly sequence. The *DUX4* gene has been previously reported to possess the most number of copies within the human genome, however the location of this gene family remains incompletely characterized. We have revealed the position of each copy within the human genome, indicating the presence of the *DUX* gene family on chromosome 3.

**
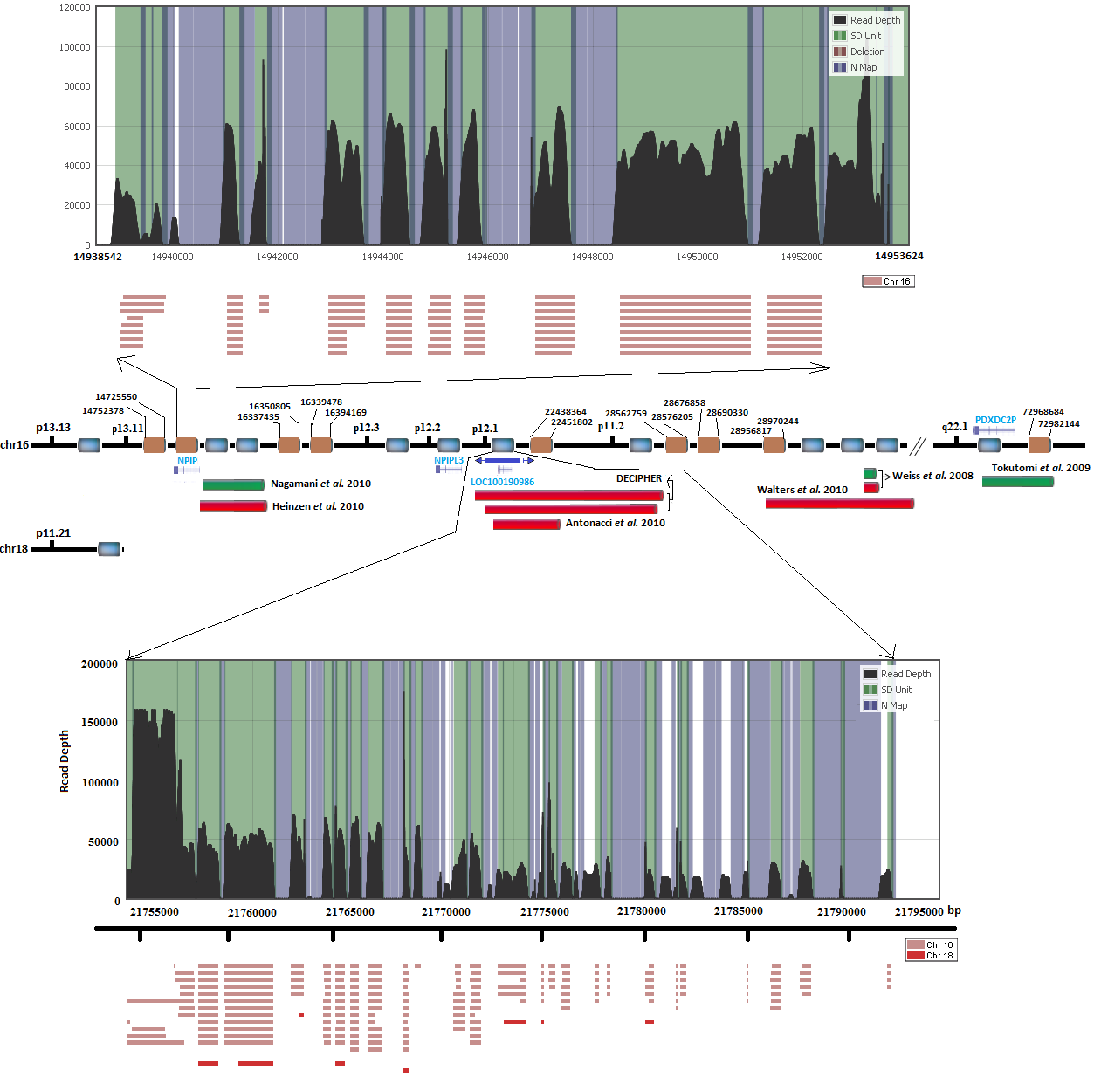
**

**Supplementary Figure 10.** Localization of the *NPIP* and *NPIPL3* gene derivatives. The alignment of the read depth plot is approximated in the chromosome contig. Multiple copies of the *NPIP* gene is located within close proximity of the *NPIPL3* gene derivatives. Our analysis revealed the localization for the *NPIP* gene is within chromosome 16, whereas the *NPIPL3* derivatives are localized in both chromosomes 16 and 18, which has been confirmed by FISH.

**Supplementary Table 2. Summary of FISH analysis.**

| **Number of cells counted** | **Chromosome modal number** | **Probes used in hybridization**  **(Probe label in brackets)** | **Chromosome hybridization of probes** | **Signal Summary**  **(approximated)** |
| --- | --- | --- | --- | --- |
| 10 | 46  10 cells with 46 chromosomes | G248P8712C10 (SpectrumOrange)  RP11-46P16 (SpectrumGreen) | Chr 1 of both homologs  Chr 1 of both homologs | Signals observed at three localizations on both chr 1 homologs:  1) at band 1p36: >one signal per homolog;  2) at band 1p10~13: poor signal;  3) at band 1q21: > multiple signals per homolog.  Two signals present, one on each homolog of chr 1 at band 1q44. |
| 10 | 46  10 cells with 46 chromosomes | G248P8661F9 (SpectrumOrange)  RP11-64L12 (SpectrumGreen) | Chr 16 and 18 of both homologs  Chr 16 of both homologs | Signals observed at three localizations on both chr 16 and chr 18 homologs:  1) at band 16p11.2~13.2: multiple signals per homolog;  2) at band 16q22: one signals on each homolog;  3) at band 18p11.2~: one signal per homolog.  Two signals present, one on each homolog of chr 16 at band 16p13.3. |
| 17 | 46  17 cells with 46 chromosomes | G248P80054G1 (SpectrumOrange)  RP11-1113I2 (SpectrumGreen) | Chr 1, 5, 20, and 22 of both homologs  Chr 22 of both homologs | Signals observed at six localizations on both chr 1, chr 5, chr 6, chr 20, and chr 22 homologs:  1) at band 1p13: one signal per homolog;  2) at band 5p13: >one signals per homolog;  3) at band 6p: >one to two signals per homolog;  4) at band 5q21: one signal per homolog.  5) at band 20p11.2 - one signal per homolog;  6) at band 22q11.2 - ~four to five signals per homolog.  Two signals present, one on each homolog of chr 22 at band 22q13. |

#
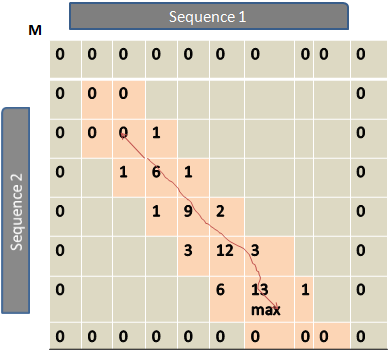


**Supplementary Figure 11.** End space free alignment algorithm. The DP matrix populates according to the cost function and only trace back occurs from the maximum position of the matrix to 0. The gap is minimized by introducing a high penalty rate (i.e., -2 or -3).

##
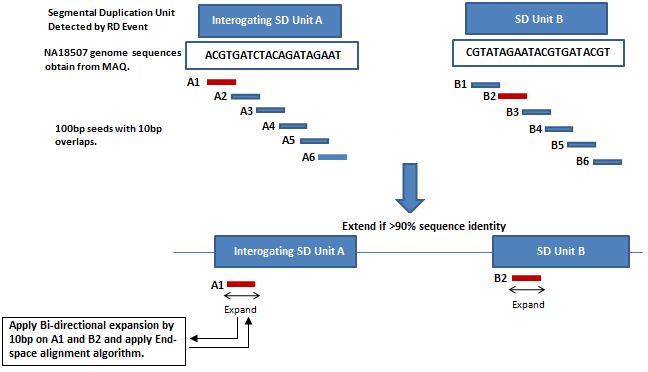


**Supplementary Figure 12.** The ‘seed and extend’ mechanism to detect optimal seeds with a 100 bp window of alignment. The seeds with <90% sequence identity were ignored for the extend step. The extend step is a recursive procedure which does not stop until it crosses the predetermined threshold of 90%. If there are multiple overlapping seeds, only seeds with maximum expansion were kept.

**Supplementary Table 3. Short reads map statistics.** Short reads were mapped using mrsFAST with an average read length of 36 bp in the NA18507 human genome with approximately 56% being mapped against the repeat masked reference genome.

| **Summary** | **Statistics** |
| --- | --- |
|  |  |
| **Genome** | NA18507 (Hapmap Yoruba sample) |
| **Platform** | Illumina Genome Analyzer |
|  |  |
| **Number of Short Reads** | 1,504,002,272 |
|  |  |
| **Total Mapped Short Reads** | 839,039,591 |

**
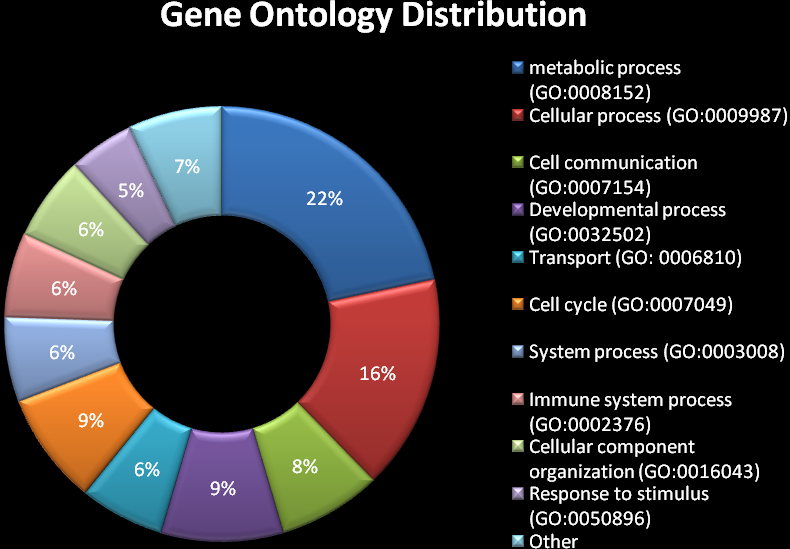
**

**Supplementary Figure 13.** Distribution of gene ontology categories for genes within rearrangement hotspot regions. The other category includes GO ID: 0006915, 0007155, 0000003, 0006091, 0042592, 0051179, 0050789.

**References**

1. Alkan C, Kidd JM, Marques-Bonet T, Antonacci F, Hormozdiari F, et al. (2009) Personalized copy number and segmental duplication maps using next- generation sequencing. Nat Genet 41: 1061-1067.

2. Conrad, D.F. Pinto, D., Redon, R., Feuk, L., Gokcumen, et al. (2010) Origin and functional impact of copy number variation in the human genome. Nature 464: 704-712.

3. Sudmant PH, Kitzman JO, Antonacci F, Alkan C, Malig M, et al. (2010) Diversity of Human Copy Number Variation and Multicopy Genes. Science 330: 641-645.
